# Deep Learner System Based on Focal Color Retinal Fundus Images to Assist in Diagnosis

**DOI:** 10.3390/diagnostics13182985

**Published:** 2023-09-18

**Authors:** Yanli Zou, Yujuan Wang, Xiangbin Kong, Tingting Chen, Jiangna Chen, Yiqun Li

**Affiliations:** 1 School of Basic Medical Sciences, Southern Medical University, Guangzhou 510000, China; ylzouzou@126.com; 2 Department of Ophthalmology, Foshan Hospital Affiliated to Southern Medical University, Foshan 528000, China; xiangbin_kong@sina.com; 3 Internal Medicine, Brookdale University Hospital Medical Center, New York, NY 11212, USA; 4 State Key Laboratory of Ophthalmology, Zhongshan Ophthalmic Center, Sun Yat-sen University, Guangzhou 510000, Chinachenjiangna@gzzoc.com (J.C.); 5 Department of Orthopedics, Foshan Hospital Affiliated to Southern Medical University, Foshan 528000, China

**Keywords:** smart healthcare system, fundus images, lesion-colored retina, auxiliary diagnosis

## Abstract

Retinal diseases are a serious and widespread ophthalmic disease that seriously affects patients’ vision and quality of life. With the aging of the population and the change in lifestyle, the incidence rate of retinal diseases has increased year by year. However, traditional diagnostic methods often require experienced doctors to analyze and judge fundus images, which carries the risk of subjectivity and misdiagnosis. This paper will analyze an intelligent medical system based on focal retinal image-aided diagnosis and use a convolutional neural network (CNN) to recognize, classify, and detect hard exudates (HEs) in fundus images (FIs). The research results indicate that under the same other conditions, the accuracy, recall, and precision of the system in diagnosing five types of patients with pathological changes under color retinal FIs range from 86.4% to 98.6%. Under conventional retinopathy FIs, the accuracy, recall, and accuracy of the system in diagnosing five types of patients ranged from 70.1% to 85%. The results show that the application of focus color retinal FIs in the intelligent medical system has high accuracy and reliability for the early detection and diagnosis of diabetic retinopathy and has important clinical applications.

## 1. Introduction

Fundus diseases are common ophthalmic diseases, including diabetic retinopathy, macular degeneration, glaucoma, etc. Accurate diagnosis and timely treatment of these diseases are crucial for protecting vision and preventing blindness. However, traditional fundus examinations rely on the experience and professional knowledge of doctors, which limits diagnostic accuracy and efficiency. With the rapid development of artificial intelligence and computer vision technology, intelligent medical systems based on lesions in color retinal fundus images are gradually becoming effective tools for improving the diagnosis of fundus diseases. The smart medical system utilizes deep learning algorithms, especially CNN, to automatically analyze and recognize lesions in fundus images. By training a large number of case image data, the system can learn the feature patterns and diagnostic rules of different diseases. When a doctor uploads a color retinal fundus image of a lesion, the system will automatically extract image features and provide corresponding diagnostic results. This way, doctors can conduct preliminary screening and diagnosis more quickly, improving the accuracy and efficiency of diagnosis.

The concept of intelligent healthcare has gradually emerged, and increasingly, scholars have conducted research on intelligent healthcare systems, resulting in a large number of achievements. Among them, intelligent healthcare utilizes new generation information technologies such as the Internet of Things (IoT), big data (BD), cloud computing, and artificial intelligence to comprehensively transform traditional healthcare systems, making healthcare more efficient, convenient, and personalized. Tian Shuo first listed the key technologies that support intelligent healthcare and introduced the current status of intelligent healthcare in several important fields, then elaborated on the problems existing in intelligent healthcare, and attempted to propose solutions. Finally, the prospects of intelligent healthcare were envisioned and evaluated [1]. Zeadally Sherali explored how IoT and BD technologies can be combined with intelligent health to provide better medical solutions, and discussed how the IoT and BD technology can be combined with intelligent health to address some challenges and improve the availability, accessibility, and cost of healthcare. The research results would help healthcare designers, professionals, and researchers design better healthcare information systems [2]. Wang Shuai proposed a parallel healthcare system framework based on artificial systems, computational experiments, and parallel execution methods. He combined emerging blockchain technology with parallel healthcare systems to build an alliance blockchain connecting patients, hospitals, health bureaus, and medical communities, achieving comprehensive medical data sharing, medical record review, and medical auditing. He built and deployed a prototype called the Parallel Gout Diagnosis and Treatment System to validate and demonstrate the effectiveness and efficiency of a blockchain-powered parallel healthcare system framework [3]. However, these studies are a single analysis of the medical system and lack research on diabetic retinopathy.

Deep learning also has many research results in the diagnosis of diabetes retinopathy. Among them, because the diagnostic criteria of diabetic retinopathy (DR) are clear, and the classification system is mature, the application of in-depth learning to diagnose diabetic retinopathy has become a research hotspot in recent years. Therefore, Fan Jiawei made a detailed review of the application of deep learning methods in the diagnosis of diabetic retinopathy from the aspects of the latest research progress of deep learning methods in DR diagnosis, the general process of DR diagnosis, public data sets, medical image annotation methods, main implementation models, and the main challenges faced so that more researchers of machine vision, especially deep learning medical images, can refer to, compare, and accelerate the maturity and clinical application of research in this field [4]. In order to evaluate the application value of the intelligent diagnostic system for screening diabetic retinopathy (DR) based on deep learning. Weng Ming collected 372 eyes of 186 patients with diabetes who visited a hospital on 6 January 2017. He compared the application of expert diagnosis and artificial intelligence diagnosis based on deep learning and compared their specificity and sensitivity. The research results indicate that the DR artificial intelligence diagnostic system based on deep learning can better display the severity of fundus lesions, and is expected to provide a new screening tool for DR [5]. The above research proves the applicability of deep learning in the diagnosis of diabetic retinopathy and provides a theoretical basis for its application in intelligent medical systems for auxiliary diagnosis.

This article will analyze the assisted diagnosis of intelligent medical systems based on lesion color retinal FIs, and introduce eye diseases, the automatic detection of HEs, and intelligent medical systems. In order to verify the relationship between focal color retinal FIs and intelligent medical system-assisted diagnosis, this article compares the diagnosis time, response time, accuracy, recall, and precision of the system under different images with patients with sugar reticulum, glaucoma, high myopia, retinal vein occlusion (RVO) and age-related macular degeneration as experimental subjects. It was found that the advent of smart medical system-assisted diagnosis based on focal color retinal FIs could provide faster and more accurate diagnostic results for physicians and more convenient and efficient medical services for patients.

## 2. Materials and Methods

### 2.1. Introduction to Eye Diseases

**Figure 1:**
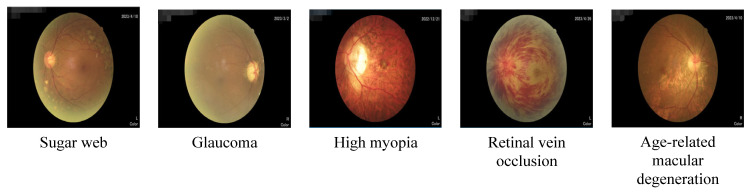
Five common eye diseases.

With the development of modern society and the change in people’s lifestyles and environmental conditions, the incidence rate of eye diseases is increasing year by year. Many eye diseases, if not treated in a timely manner or treated improperly, may lead to serious visual impairment and even blindness. Therefore, it is essential to prevent eye disease, conduct regular eye examinations, and receive timely treatment. Sugar web, glaucoma, high myopia, RVO, and age-related macular degeneration are all common eye diseases, as shown in Figure 1.

Diabetic patients are prone to diabetic retinopathy, that is, sugar web, and sugar web is one of the important causes of blindness. Diabetic retinopathy is one of the most common ocular complications in diabetic patients. Long-term high blood sugar levels cause damage to blood vessels in the eyes, leading to the occurrence of retinopathy. Diabetic retinopathy can be divided into two types: non-proliferative and proliferative.

Non-proliferative diabetic retinopathy includes microangioma, microvascular blockage, and leakage. Microhemangioma is the dilation and embrittlement of blood vessels, which are prone to rupture and bleeding; microvascular occlusion is stenosis or occlusion, resulting in retinal ischemia; and leakage is an increase in the permeability of the blood vessel wall, allowing fluid and proteins to seep into the retinal tissue.

Proliferative diabetic retinopathy refers to the growth of new blood vessels based on non-proliferative diseases. These new blood vessels are fragile and prone to rupture, which may lead to bleeding and retinal detachment. If the condition progresses severely, it may lead to severe visual impairment or even blindness.

Early diabetic retinopathy usually has no obvious symptoms. With the development of the disease, patients may have blurred vision, visual field loss, dark adaptation, ability decline, and other symptoms. Regular fundus examination is an important means to prevent and detect early diabetic retinopathy.

The methods of treating diabetic retinopathy include controlling blood sugar levels, controlling blood pressure, injecting anti-antigenic drugs, laser therapy, and surgical treatment. Early diagnosis and treatment can effectively slow down the progression of lesions and protect vision. Therefore, patients with diabetes should have regular eye examinations and actively manage diabetes to prevent and control the occurrence and progress of diabetic retinopathy.

Glaucoma is a disease caused by high intraocular pressure. If not treated in a timely manner, it can lead to a serious decline in vision and seriously affect the quality of life. Color fundus images are one of the commonly used tools for diagnosing glaucoma. Although color fundus images cannot directly detect glaucoma, they can provide important information that helps with glaucoma screening and diagnosis.

Color fundus images are considered a sufficient marker for glaucoma, mainly for the following reasons:

Glaucoma is a group of diseases with the common characteristics of optic papillary atrophy and depression, visual field defect, and vision loss. Pathological increase in intraocular pressure and insufficient optic nerve blood supply are the primary risk factors. The optic nerve tolerance to pressure damage is also related to the occurrence and development of glaucoma. Color fundus images can visually display the condition of the fundus, including the optic disc, retina, and blood vessels. By observing fundus images, doctors can evaluate the shape and color of the optic disc, as well as the status of the retina and blood vessels to determine whether there is damage caused by glaucoma.

Color fundus images can help doctors detect early lesions in glaucoma. Early glaucoma lesions may not show obvious symptoms, but fundus images can display subtle changes such as deformation of the optic disc, color changes, retinal damage, etc., providing the possibility of early diagnosis.

Color fundus imaging is a non-invasive and painless examination method that is more convenient and comfortable for patients. By capturing fundus images, a large amount of information can be quickly obtained, reducing patients’ discomfort and inconvenience.

It should be noted that although color fundus images can serve as a marker for glaucoma, comprehensive judgment still needs to be made in conjunction with other clinical manifestations and examination results. Only the professional diagnosis of a doctor can determine whether one has glaucoma. If you suspect that you may have glaucoma, you should promptly consult a professional doctor for diagnosis and treatment.

High myopia is a serious myopia with a degree of myopia exceeding 600 degrees. If left untreated for a long time, complications such as retinal detachment may also occur.

RVO is a disease caused by retinal vascular occlusion, mainly manifested as sudden loss of vision or blindness, which requires timely treatment to avoid serious consequences.

Senile macular degeneration is a common degenerative eye disease that often occurs in the eyes of elderly people. Macular degeneration can lead to decreased central vision, which seriously affects the quality of life. At present, the main methods for treating age-related macular degeneration include laser therapy and surgical treatment.

Although color fundus photographs have been widely used in the detection of diabetic retinopathy, they still need the interpretation and judgment of professional doctors. Therefore, if people have diabetes or a family history of diabetes, it is recommended to conduct a fundus examination regularly to detect and treat diabetic retinopathy as soon as possible.

### 2.2. HEs Automatic Detection

HEs are the most prominent fundus lesions in the early stages of glucose reticulopathy and their automatic identification is a key aspect of the supplementary diagnostic system for glucose reticulopathy. Therefore, efficient automatic recognition of HEs for patients with diabetic retinopathy is of great clinical significance for its large base and high incidence rate. Currently, the automatic detection of HEs is mostly based on machine learning methods, which select a set of features based on experience, establish a classifier, and then use shallow learning methods to locate and recognize HEs in FIs.

In recent years, deep learning algorithms represented by CNN have made significant breakthroughs in image processing. Compared with shallow learning, deep learning exhibits many advantages, as it does not require manual setting of features to be extracted and has strong generalization ability. Therefore, this article will use deep learning technology to conduct deep feature learning on HEs and establish a classification model for HEs.

(1) Convolutional neural network

CNN (Convolutional neural network) is an algorithm for deep learning. Deep learning is a machine learning method that learns and trains via multi-layer neural networks. As a special type of neural network for deep learning, CNN is mainly used to process data with spatial structure, such as images, speech, etc.

CNN extracts features from images and classifies them via components such as convolutional layers, pooling layers, and fully connected layers. The convolutional layer extracts local features of the image via convolution operations; the pooling layer reduces the dimensionality of features via downsampling operations; and the fully connected layer performs tasks such as classification or regression via multiple neurons. The network structure of CNN can be adjusted and designed based on the complexity of tasks and the characteristics of data.

CNN is a deep learning method that can automatically learn features from images and achieve excellent performance in tasks such as classification and recognition. In smart medical systems based on color retinal FIs of lesions, CNN can be used as a tool for image recognition and classification, automatically detecting and diagnosing important information such as the location, size, and type of lesions, and providing corresponding diagnostic results and recommendations. Therefore, the application of CNN in the intelligent medical system to focus color retinal FIs can greatly improve the early diagnosis and treatment of eye diseases such as diabetic retinopathy. For smart medical system-aided diagnosis based on color retinal FIs of lesions, CNN can help ophthalmologists quickly and accurately locate and identify lesion lesions.

When using CNN for the recognition and analysis of lesion color retinal FIs, the image is first input into a convolutional layer and filtered via convolutional verification to extract a series of feature maps. Next, downsampling is performed in the pooling layer to reduce the size and complexity of the feature map while preserving the most significant features. Finally, features are classified and predicted in the fully connected layer.

The input layer is a two-dimensional sample image, and each convolution layer is composed of multiple feature maps. The feature map $A_{o}^{z-1}$ obtained from the previous layer is convolved with the learnable convolution kernel $l_{ok}^{z}$, and the convolution result is generated into feature map $A_{k}^{z}$ via the nonlinear activation function $g\left(.\right)$. The specific form is as follows:

(1) $$A_{k}^{z}=g(\sum_{o\in N_{k}}A_{o}^{z-1}*l_{ok}^{z}+y_{k}^{z})$$

In the formula, $A_{k}^{z}$ represents the output of the k-th feature map of layer 1; $y_{k}^{z}$ corresponds to the offset of $A_{k}^{z}$; ∗ represents convolution operation; $l_{ok}^{z}$ is a convolutional kernel that can be convolved with multiple feature maps of layers (1–1); and $N_{k}$ represents the set of input feature maps corresponding to $A_{k}^{z}$. The commonly used activation functions of CNN include the Sigmaid function (Formula (2)), Tanh function (Formula (3)), ReLU function (Formula (4)), etc., which can simulate the response of neurons to excitation.

(2) $$g_{d}\left(A\right)=\frac{1}{1+r^{-a}}$$

(3) $$g_{d}\left(A\right)=tanh\left(a\right)$$

(4) $$g_{d}\left(A\right)=max\left(a,0\right)$$

The sampling layer implements the downsampling processing of each feature map. The common methods in this layer are pooling the maximum value and pooling the average value. The expressions of the two are

(5) $$A_{k}^{z}=g\left(\underset{o\in m*m}{\max}A_{k}^{z-1}i\left(m,m\right)\right)$$

(6) $$A_{k}^{z}=g\left(\frac{1}{m*m}\right)\sum_{o\in m*m}A_{k}^{z-1}i\left(m,m\right)$$

In the formula, $i\left(m,m\right)$ is the window function input for the sampling layer. Finally, the fully connected layer performs regression, classification, and other processing on the previously extracted features via layer-by-layer transformation and mapping. Like the output layer, the trained feature maps are summarized into feature vectors to train the HE classification model. CNN is suitable for processing complex data such as high-dimensional data and images, and can help doctors quickly and accurately locate and identify various lesions, such as macular holes, edema, frontal disc melanoma, etc. Therefore, there is a close relationship between CNN and color retinal FIs of lesions, which is an important component of diagnostic technology in smart medical systems.

(2) Evaluation indicators

Currently, there are two main performance evaluations for HE automatic recognition algorithms: one is based on image level evaluation indicators, and the other is based on lesion area evaluation indicators. There are three main evaluation indicators for evaluating automatic detection methods: accuracy, recall, and precision. The calculation formulas are as follows:

(7) $$\begin{array}{c} Precision=\frac{TP}{TP+FP}\\ Recall=\frac{TP}{TP+FN}\\ Accuracy=\frac{TP+TN}{TP+TN+FP+FN} \end{array}$$

In Formula (8), TP, FP, FN, and TN are true positive, false positive, false negative, and true negative, respectively. In the image-based assessment index, the unit of this assessment index is the number of whole FIs, whereas in the lesion level-based assessment index, the unit of this assessment index is the number of areas. This index reflects the probability of hard exudate areas appearing in FIs and can better evaluate the effectiveness of the algorithm.

### 2.3. Smart Medical System

The smart medical system is an advanced medical service system that utilizes technologies such as artificial intelligence, BD, and cloud computing to achieve medical informatization and intelligence [6,7]. It can achieve disease diagnosis, treatment, and prevention more quickly and accurately via the effective integration and analysis of medical data, knowledge, and experience.

Smart medical systems can be applied to multiple medical fields, such as imaging, diagnosis, remote medicine, intelligent monitoring, drug research and development, etc. [8,9]. Among them, medical image diagnosis systems based on BD and artificial intelligence technology have become an important branch of smart medical systems. It can automatically detect, classify, and locate different medical images, improving the work efficiency and diagnostic accuracy of doctors [10,11]. In addition, smart healthcare systems can also achieve the sharing and allocation of medical resources by connecting hospitals, doctors, and patients. Compared with traditional healthcare, they have advantages such as more convenience, efficiency, low cost, and sustainability.

**Figure 2:**
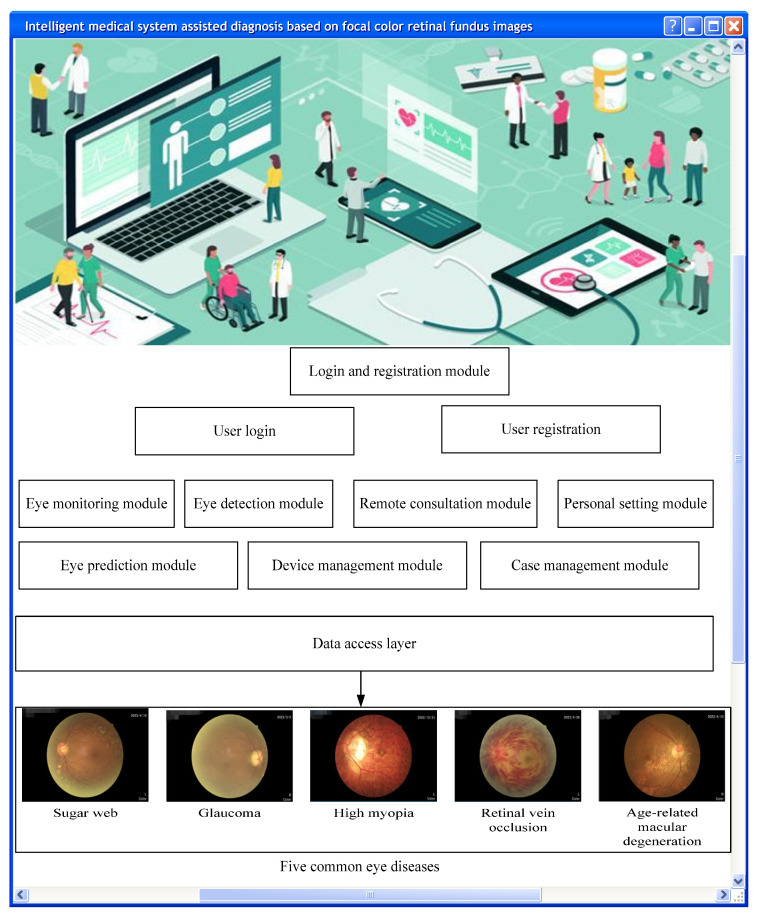
Logical architecture of the intelligent medical system.

In short, smart medical systems are medical service systems with broad application prospects that integrate medical informatization and intelligence, providing patients with more convenient, efficient, and accurate medical services [12,13]. The system’s logical architecture design is shown in Figure 2.

Color retinal FIs of lesions are one of the important criteria for ophthalmologists to diagnose and treat diseases. They can directly reflect information such as eye tissue structure and pathological changes and therefore have essential clinical value. The smart medical system is a new technology system for the diagnosis and treatment of ophthalmic diseases. By integrating and applying advanced computer technology and data processing algorithms, it provides accurate, fast, and intelligent diagnosis, detection, and analysis services for ophthalmologists [14,15].

In smart medical systems, color retinal FIs of lesions are an important component [16]. By utilizing computer vision and artificial intelligence technology to analyze these images, automated disease diagnosis and auxiliary decision making can be achieved, improving diagnostic accuracy and efficiency. The emergence of such smart medical systems would greatly help the work of ophthalmologists by alleviating the shortage of physician resources and improving the efficiency and accuracy of medical care, which is expected to bring new opportunities and challenges for the treatment and prevention of ophthalmic diseases [17,18].

## 3. Results

### 3.1. Experimental Preparation

(1) Experimental purpose

The diagnostic accuracy and usefulness of focal color retinal FIs and focal conventional retinal FIs for smart medical systems and comparison with physician diagnosis were evaluated.

(2) Experimental subjects

This article selected patients who were diagnosed with diabetic retinopathy, glaucoma, high myopia, RVO, or age-related macular degeneration. There were 20 patients of each type, and then 10 normal samples were added, with a total sample size of 150.

(3) The experimental environment was a real clinical environment such as a hospital ophthalmology clinic.
(4) Experimental design:

Step one is to collect the color retinal FIs of the patient’s lesion and input them into the intelligent medical system for automatic diagnosis, recording the diagnostic results and time of each.

Step two is to collect the routine retinal FIs of the patient’s lesion, input them into the intelligent medical system for automatic diagnosis, and record the diagnostic results and time of each.

Step three is for the doctor to re-examine and diagnose the patient based on the system diagnostic results, and record the diagnostic results.

Step four is to compare and analyze the diagnostic results of the smart medical system using lesion color retinal FIs, the diagnostic results of the smart medical system using lesion conventional retinal FIs, and the final diagnostic results of the doctor, in order to evaluate the auxiliary diagnostic effect of lesion color retinal FIs on the smart medical system.

(5) Experimental indicators

Diagnostic time refers to the diagnostic time of the system when using different images.

Response time: Response time refers to the time from image input to the start of system diagnosis. In practical applications, the shorter the reaction time, the better the system can meet the needs of clinical work.

Accuracy is the proportion of the system’s diagnostic results consistent with the doctor’s final diagnostic results under different images.

The recall rate is the ratio between the number of lesions detected by the system in different images and the number of lesions in the doctor’s final diagnosis.

Precision is the consistency between the location and size of lesions detected by the system in different images and the final diagnosis result of the doctor.

### 3.2. Experimental Results

In this paper, patients with Tangwang glaucoma, high myopia, retinal vein occlusion, and senile macular degeneration were first designed as a group, and a total of 10 groups were prepared. Then, the focus color retinal fundus images and the focus conventional retinal fundus images of each group of patients were input into the intelligent medical treatment system for automatic diagnosis, and the total time required for diagnosis and total reaction time were recorded; then, the diagnostic results with the doctor’s final diagnostic results were compared, and the average accuracy, recall, and accuracy were calculated; finally, for the rigor of the experiment, the number of diagnoses in this article was set to 5.

(1) Total time required for diagnosis

**Figure 3:**
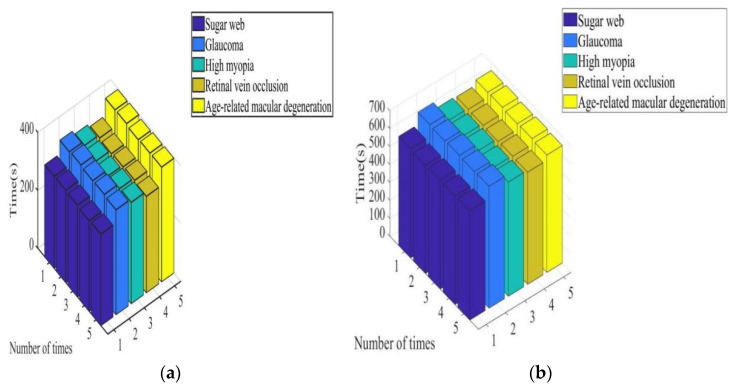
Total time required for system diagnosis under different images. (**a**) Color retinal FIs of lesions and (**b**) conventional retinal FIs of lesions.

In Figure 3a, it can be seen that the total time required for the system to diagnose patients with diabetic retinopathy, glaucoma, high myopia, RVO, and age-related macular degeneration was between 311–320 s, 360–369 s, 340–350 s, 330–339 s, and 390–398 s, respectively. In Figure 3b, it can be seen that the total time required for the system to diagnose patients with diabetic retinopathy, glaucoma, high myopia, RVO, and age-related macular degeneration was between 601–610 s, 670–679 s, 630–640 s, 621–629 s, and 652–660 s, respectively. In Figure 3, it can be seen that under the color retinal FI of the lesion, the total time required for the system to diagnose the five types of patients was between 311 s and 398 s. Under conventional retinal FIs of lesions, the total time required for the system to diagnose the five types of patients was between 601 s and 679 s. It is shown that focal color retinal FIs can reduce the time required for diagnosis aided by intelligent medical systems.

(2) Total reaction time

**Figure 4:**
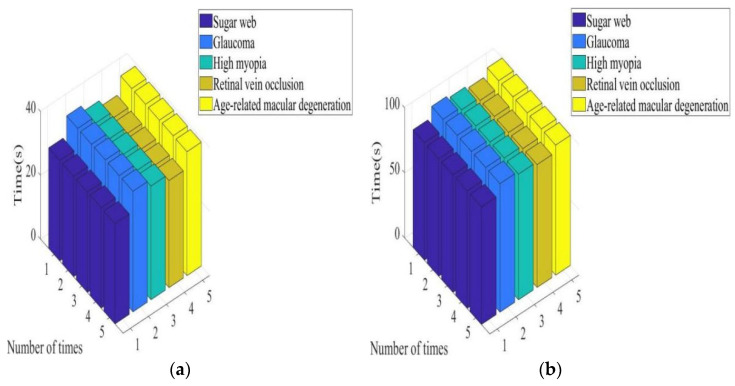
Total response time required for system diagnosis under different images. (**a**) Color retinal FIs of lesions and (**b**) conventional retinal FIs of lesions.

In Figure 4a, it can be seen that the total response time required for the diagnosis of patients with diabetic retinopathy, glaucoma, high myopia, RVO, and age-related macular degeneration by the system was between 31.41–31.5 s, 37.71–37.8 s, 35.61–35.7 s, 33.52–33.59 s, and 38.81–38.9 s, respectively. In Figure 4b, it can be seen that the total response time required for the diagnosis of patients with diabetic retinopathy, glaucoma, high myopia, RVO, and age-related macular degeneration by the system was between 90.22–90.3 s, 98.72–98.8 s, 96.52–96.6 s, 94.3–94.4 s, and 99.91–99.99 s, respectively. In Figure 4, it can be seen that under the color retinal FI of the lesion, the total response time required for the system to diagnose the five types of patients was between 31.41 and 38.9 s. Under conventional retinal FIs of lesions, the total response time required for the system to diagnose the five types of patients was between 90.22 and 99.99 s. It is shown that focal color retinal FIs can optimize the response time when aided in diagnosis by intelligent medical systems.

(3) Accuracy

**Figure 5:**
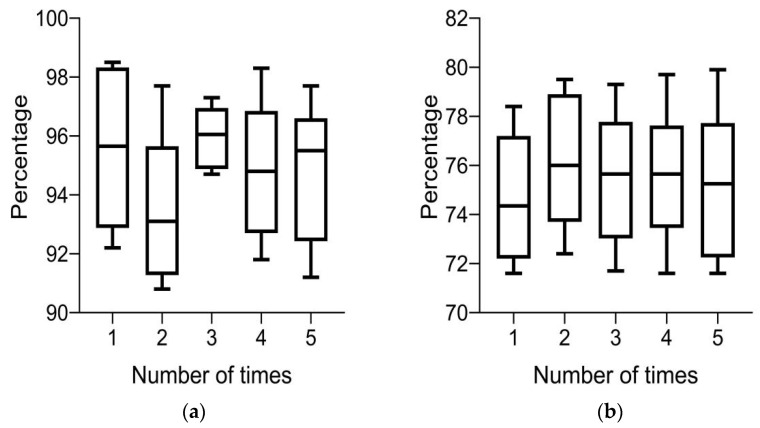
Accuracy of system diagnosis under different images. (**a**) Color retinal FIs of lesions and (**b**) conventional retinal FIs of lesions.

In Figure 5a, it can be seen that the accuracy of the system’s five rounds of color retinal fundus images for 10 groups of lesions is above 90%; in Figure 5b, it can be seen that the accuracy of the system’s routine retinal fundus images for 10 groups of lesions is between 70 and 80% five times; and in Figure 5, it can be seen that the system has a higher accuracy for color retinal fundus images of lesions. These results indicate that color retinal fundus images of lesions can improve the accuracy of intelligent medical system-assisted diagnosis.

(4) Recall rate

**Figure 6:**
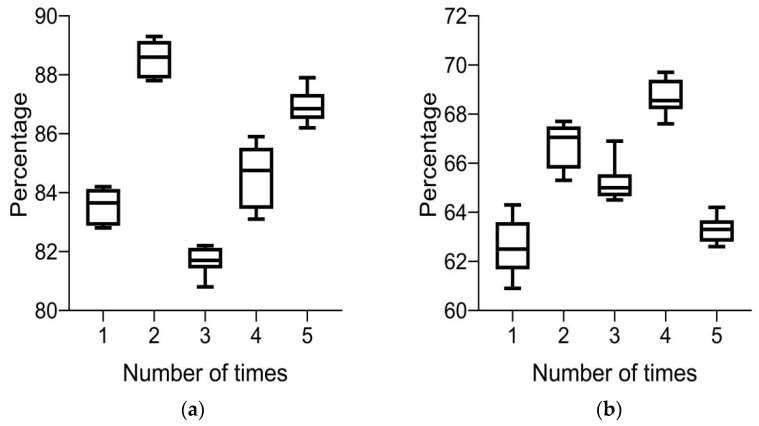
Recall rate of system diagnosis under different images. (**a**) Color retinal FIs of lesions and (**b**) conventional retinal FIs of lesions.

In Figure 6a, it can be seen that the recall rates of the system for color retinal fundus images of 10 groups of lesions are between 80 and 90% five times; in Figure 6b, it can be seen that the recall rates of the system for routine retinal fundus images of 10 groups of lesions are between 60 and 70% five times; and in Figure 6, it can be seen that the system has a higher recall rate for color retinal fundus images of lesions. These results indicate that color retinal fundus images of lesions can improve the recall rate of intelligent medical system-assisted diagnosis.

(5) Precision

**Figure 7:**
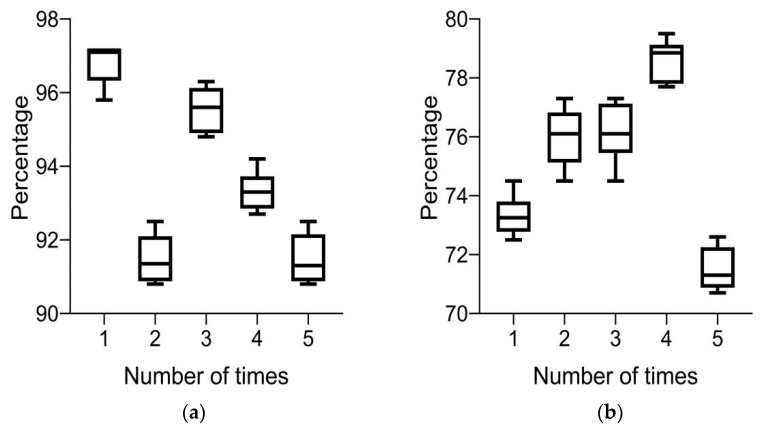
Precision of system diagnosis under different images. (**a**) Color retinal FIs of lesions and (**b**) conventional retinal FIs of lesions.

In Figure 7a, it can be seen that the accuracy of the system’s five rounds of color retinal fundus images for 10 groups of lesions is above 90%; in Figure 7b, it can be seen that the accuracy of the system’s routine retinal fundus images for 10 groups of lesions is between 70 and 80% five times; and in Figure 7, it can be seen that the system has a higher accuracy for color retinal fundus images of lesions. These results indicate that color retinal fundus images of lesions can improve the accuracy of intelligent medical system-assisted diagnosis.

## 4. Discussion

In summary, under the color retinal fundus images of lesions, the accuracy, recall, and accuracy of the system in diagnosing 10 groups of patients each time are above 80%; and under routine retinal fundus images of lesions, the accuracy, recall, and accuracy of the system for diagnosing 10 groups of patients each time ranged from 60% to 80%, Indicating a positive relationship between color retinal fundus images of lesions and intelligent medical system-assisted diagnosis. It should be noted that the results of the system’s automatic diagnosis need to be evaluated and confirmed by doctors to ensure the accuracy and reliability of the diagnosis. Therefore, in practical applications, it is necessary to combine the advantages and disadvantages of system automatic diagnosis, as well as the actual work needs and professional knowledge of doctors, and comprehensively consider multiple factors to achieve the best clinical application effects.

## 5. Conclusions

This article mainly studies the relationship between color retinal fundus images of lesions and intelligent medical systems. This study indicates that a smart medical system based on lesion color retinal fundus images can effectively assist in the diagnosis of eye diseases. By analyzing lesions in color retinal images, the system can provide fast and accurate diagnostic results. Compared to conventional retinal images, smart medical systems have higher efficiency and accuracy in color retinal images. In addition, it can also help the system automatically identify and track the development of lesions, helping doctors develop more reasonable treatment plans. Therefore, intelligent medical systems based on lesion color retinal fundus images have broad application prospects in ophthalmic diagnosis.

## Data Availability

No data were used to support this study.
